# Brief Educational Video plus Telecare to Enhance Recovery for Older Emergency Department Patients with Acute Musculoskeletal Pain: an update to the study protocol for a randomized controlled trial

**DOI:** 10.1186/s13063-022-06310-z

**Published:** 2022-05-12

**Authors:** Karen Hurka-Richardson, Timothy F. Platts-Mills, Samuel A. McLean, Morris Weinberger, Sally C. Stearns, Montika Bush, Eugenia Quackenbush, Srihari Chari, Aileen Aylward, Kurt Kroenke, Robert D. Kerns, Mark A. Weaver, Francis J. Keefe, David Berkoff, Michelle L. Meyer

**Affiliations:** 1 Department of Emergency Medicine, University of North Carolina at Chapel Hill, Houpt Bldg, 170 Manning Dr, Chapel Hill, NC USA; 2 Ophirex, Inc., Corte Madera, CA USA; 3 Department of Anesthesiology, University of North Carolina Hospitals, Chapel Hill, NC USA; 4 Department of Health Policy and Management, Gillings School of Global Public Health, University of North Carolina at Chapel Hill, Chapel Hill, NC USA; 5 Regenstrief Institute and Department of Medicine, Indiana University, Indianapolis, IN USA; 6 Departments of Psychiatry, Neurology and Psychology, Yale University, New Haven, CT USA; 7 Department of Mathematics and Statistics, Elon University, Elon, NC USA; 8 Department of Psychology and Neuroscience, Duke University, Durham, NC USA; 9 Department of Orthopedics, University of North Carolina at Chapel Hill, Chapel Hill, NC USA

**Keywords:** Musculoskeletal pain, Geriatrics, Emergency medicine, Shared decision-making

## Abstract

**Background:**

This update describes changes to the Brief Educational Tool to Enhance Recovery (BETTER) trial in response to the COVID-19 pandemic.

**Methods/design:**

The original protocol was published in *Trials*. Due to the COVID-19 pandemic, the BETTER trial converted to remote recruitment in April 2020. All recruitment, consent, enrollment, and randomization now occur by phone within 24 h of the acute care visit. Other changes to the original protocol include an expansion of inclusion criteria and addition of new recruitment sites. To increase recruitment numbers, eligibility criteria were expanded to include individuals with chronic pain, non-daily opioid use within 2 weeks of enrollment, presenting musculoskeletal pain (MSP) symptoms for more than 1 week, hospitalization in past 30 days, and not the first time seeking medical treatment for presenting MSP pain. In addition, recruitment sites were expanded to other emergency departments and an orthopedic urgent care clinic.

**Conclusions:**

Recruiting from an orthopedic urgent care clinic and transitioning to remote operations not only allowed for continued participant enrollment during the pandemic but also resulted in some favorable outcomes, including operational efficiencies, increased enrollment, and broader generalizability.

**Trial registration:**

ClinicalTrials.gov NCT04118595. Registered on October 8, 2019.

## Introduction

The Brief Educational Video plus Telecare to Enhance Recovery for Older Emergency Department Patients with Acute Musculoskeletal Pain (BETTER) is a 3-arm, randomized controlled clinical trial (RCT) comparing an educational video and telecare vs. video only vs. usual care for preventing the transition from acute to chronic musculoskeletal pain (MSP) among older adults in emergency department (ED) settings. This update describes changes made to the BETTER protocol [1] in response to low recruitment numbers and the COVID-19 pandemic. Changes include broadening inclusion criteria to boost enrollment numbers, opening recruitment to additional acute care sites, and transitioning to remote operations due to suspension of in-person research activities in the spring of 2020 resulting from the COVID-19 pandemic.

## Changes to inclusionary criteria and expansion to new sites

After receiving institutional review board (IRB) approval, recruitment commenced in the University of North Carolina (UNC) Medical Center ED in February 2020. Initially, subjects were excluded from the trial for the following reasons: (1) critical illness, (2) hospital admission, (3) dementia, (4) nursing home resident or homelessness, (5) opioid use in the past 3 months, (6) non-English speaking, (7) on psychiatric hold or excluded psych diagnosis (bipolar disorder, schizophrenia, substance abuse), (8) unable to consent (e.g., hearing loss, blindness), (9) pain more than 1 week prior to ED visit, (10) not the first time seeking medical care for complaint, (11) hospital admission in past 30 days, (12) chronic pain, (13) prisoner, and (14) pain due to ischemia, infection, kidney stone, and cancer (not due to MSP). However, enrollment was hampered by these strict eligibility requirements, in particular limiting recruitment to acute MSP, as many older adults also suffer from chronic MSP. After consulting with study co-investigators and receiving IRB approval, the protocol was updated with expanded inclusion criteria. In March 2020, eligibility was expanded to include subjects with opioid use in the past 3 months (but excluded patients reporting daily opioid use for more than 2 weeks), presenting MSP for more than 1 week prior to the ED visit, prior treatment for their presenting MSP complaint, hospitalization in past 30 days, and ongoing chronic pain (pain which lasts 3 months or more on most days). In addition, because many older adults presenting to the ED with acute or chronic MSP are admitted to the hospital (making them ineligible to participate in the study), UNC OrthoNow, an orthopedic urgent care clinic (UCC) in Chapel Hill, NC, was added as a recruitment site.

In April 2020, UNC suspended in-person research activities for all studies except those evaluating a potential life-saving intervention. The trial subsequently transitioned to fully remote operations including recruitment, consent, enrollment, and video/telecare interventions. Converting the study to remote operations has had the benefit of allowing recruitment from more study sites, as one research assistant can monitor multiple sites from one location by checking the electronic medical records at all three participating EDs (UNC, REX, Hillsborough) and OrthoNow UCC. Recruitment, telecare, and follow-up phone calls are all completed remotely using cellular phones encrypted by UNC Information Technology for secure communication. The UNC IRB granted permission to obtain the Health Insurance Portability and Accountability Act of 1996 (HIPAA) authorization and consent verbally without signatures; however, subjects are provided with an electronic or paper copy of both forms for their records.

Subjects randomized to watch the video are texted or emailed a link to the video and have 24 h from discharge to confirm via email, text, or phone that they have watched the video. Due to technical challenges and privacy concerns, telecare calls are not recorded, but the duration of each call is tracked and there is one research nurse to ensure consistency with the telecare script. While remote operations have largely been successful in this trial, one important limitation involves the shared decision-making component described in our previously published protocol. Because the video intervention cannot be delivered in the clinical setting, the intervention does not have the potential to inform conversations and decision-making during the clinical visit. Accordingly, we have removed the 9-Item Shared Decision-Making Questionnaires from our follow-up phone calls.

As of October 29, 2021, 260 subjects have enrolled in the study. We initially estimated that randomizing 360 patients would provide at least 90% power for testing the overall null hypothesis of no difference across the three arms. This calculation assumed loss-to-follow-up would be exponentially distributed such that the total loss would be 10% at 6 months. However, our current retention through 6 months is greater than 96%. Allowing for up to 5% loss through 6 months, with all other assumptions from the original calculations held the same, we find that randomizing 110 patients to each arm would still provide at least 90% power for testing the overall null hypothesis. Thus, to account for setbacks in enrollment due to the COVID-19 pandemic, we will adjust our total target sample size to at least 330. We estimate meeting our enrollment goal by June 2022.

It is important to note that this study was originally designed to be implemented in the ED; however, most of the participants in this trial are being enrolled from the orthopedic UCC clinic. Recruiting older adults seen in EDs has been challenging for our team because this population tends to be sicker than UCC patients and is frequently admitted to the hospital, which is an exclusion criterion for this study. Additionally, patients discharged from the ED often do not respond to calls or, if they do respond, usually decline participation, perhaps due to lingering physical and emotional stressors related to their ED visit.

During the study, EDs experienced a drop in patient volume, possibly related to fear of exposure to COVID-19, thus recruiting from the orthopedic UCC has allowed us to continue participant enrollment during the pandemic and has resulted in some favorable outcomes, including improving operational efficiencies and increasing the number of patients to screen and enroll. Another benefit of expanding to an orthopedic UCC is increased generalizability of the findings to older adults with MSP presenting to a variety of treatment settings. Many older adults presenting either physically to primary care, urgent care, or retail clinics, or remotely via video, stand to benefit from these interventions, expediting their recovery time and reducing the risk of developing chronic pain.

## Data Availability

Deidentified data will be shared on ClinicalTrials.gov 9 to 36 months following publication. Investigators who would like access to the full dataset may contact the PI directly.

## Abbreviations

BETTER: Brief Educational Tool to Enhance Recovery
ED: Emergency department
MSP: Musculoskeletal pain
UC: Urgent care

## References

[1] Platts-Mills TF, McLean SA, Weinberger M, Stearns SC, Bush M, Teresi BB, Hurka-Richardson K, Kroenke K, Kerns RD, Weaver MA, Keefe FJ (2020). Brief educational video plus telecare to enhance recovery for older emergency department patients with acute musculoskeletal pain: study protocol for the BETTER randomized controlled trial. Trials.

